# Valproic Acid Increases the Hepatic Differentiation Potential of Salivary Gland Cells

**Published:** 2015

**Authors:** O. S. Petrakova, V. V. Ashapkin, V. Y. Shtratnikova, L. I. Kutueva, E. A. Vorotelyak, M. A. Borisov, V. V. Terskikh, I. G. Gvazava, A. V. Vasiliev

**Affiliations:** Lomonosov Moscow State University, Faculty of Biology, Leninskie Gory, Moscow State University, 1, bld. 12, 119991, Moscow, Russia; Pirogov Russian National Research Medical University, Ostrovitianov str., 1, 117997, Moscow, Russia; Belozersky Institute, Moscow State University, Leninskie Gory, 1/40, 119991, Moscow, Russia; Department of bioengineering and bioinformatics, Lomonosov Moscow State University, Leninskie Gory, 1/73, 119991, Moscow, Russia; Koltsov Institute of Developmental Biology, Russian Academy of Sciences, Vavilova str., 26, 119334 , Moscow, Russia

**Keywords:** gene expression, hepatic differentiation, submandibular salivary gland cells, valproic acid

## Abstract

The studies of cell plasticity and differentiation abilities are important problems in modern cellular biology. The use of histone deacetylase inhibitor - valproic acid is a promising approach to increasing the differentiation efficiency of various cell types. In this paper we investigate the ability of mouse submandibular salivary gland cells to differentiate into the hepatic direction and the effect of valproic acid on the efficiency of this differentiation. It was shown that the gene expression levels of hepatocyte markers (*Aat*, *Afp*, *G6p*, *Pepck*,* Tat*, *Cyp3a13*) and liver-enriched transcription factors (*Hnf-3α*, *Hnf-3β*, *Hnf-4α*, *Hnf-6*) were increased after differentiation in salivary gland cells. Valproic acid increases the specificity of hepatic differentiation, reducing the expression levels of the ductal (*Krt19*, *Hhex1*, *Cyp7a1*) and acinar (*Ptf1a*) markers. After valproic acid exposure, the efficiency of hepatic differentiation also increases, as evidenced by the increase in the gene expression level of *Alb *and *Tdo*, and increase in urea production by differentiated cells. No change was found in DNA methylation of the promoter regions of the genes; however, valproic acid treatment and subsequent hepatic differentiation largely affected the histone H3 methylation of liver-enriched genes. Thus, mouse submandibular salivary gland cells are capable of effective differentiation in the hepatic direction. Valproic acid increases the specificity and efficiency of the hepatic differentiation of these cells.

## INTRODUCTION

The investigation of cell differentiation abilities is one of the most important problems in modern cell biology.* In vitro *expansion and subsequent differentiation can provide cells for regenerative medicine, as well as help in the study of development regulation and drug discovery. One of the promising approaches in this field is the use of small molecules possessing the ability to change the epigenetic status of cells [1]. Using small molecules facilitates cell reprogramming and increases the differentiation efficiency of various cell types [2-5].

Valproic acid (valproate, 2-*n*-propylpentanoic acid, VPA) has been used for decades as an effective antiepileptic drug with a broad spectrum of action [6]. Valproic acid acts as an inhibitor of histone deacetylases thereby causing an increase in gene expression [7]. The idea of histone deacetylase inhibitors application is based on the fact that histone acetylation causes the activation of various gene expressions, resulting in an increase in the transcription pool of cells and, hence, increases the cell differentiation ability. Some researchers believe that this effect on cell epigenetic regulation may also cause cells dedifferentiation [8].

With regard to the endoderm, liver and pancreas are two crucial organs of this germ layer. The ability of valproic acid to improve the efficiency of hepatic differentiation both in pluripotent and differentiated cells has been shown [9, 10]. The use of valproic acid during a standard hepatic differentiation protocol increases the hepatocyte differentiation of mouse embryonic stem (ES) cells and reduces the differentiation into ductal structures [9]. Human bone marrow cells much more efficiently differentiate in the hepatic direction after 5mM valproic acid exposure. These cells express albumin and produce urea more efficiently than cells not exposed to valproic acid treatment [10]. Human umbilical-cord-derived mesenchymal stem cells more efficiently undergo hepatic differentiation after 10mM valproic acid exposure in a concentration-dependent manner [11].

Cellular therapies of liver and pancreas disorders are hampered by a limited source of cells with the ability to differentiate within endoderm with high efficiency. The search for cells capable of differentiation in the hepatic and pancreatic directions with high efficiency is a challenge. Submandibular salivary gland cells have high differentiation abilities within endoderm and are an attractive source of adult cells for the cellular therapy of liver and pancreas disorders [12-14]. Ductal epithelial cells of submandibular salivary glands are easily accessible from patients and are easy to culture. As it was shown previously, submandibular salivary gland cells (SGC) isolated from humans and different animals (mouse, rat and swine) represent an active proliferating culture *in vitro *and possess high differentiation ability in the hepatic and pancreatic directions [13, 15-17]. Cultured submandibular salivary gland cells possess phenotypic convergence with liver progenitor cells (LPS) in mice [18]. A comparative *in vitro *analysis of mouse submandibular salivary gland and liver progenitor cells revealed similarities in cell markers gene expression: EpCAM, CD29, c-Kit, Sca-1, c-Met, cytokeratins 8, 18, 19, Afp, as well as in regulatory factors gene expression [18]. Under certain conditions, SGC acquire the ability of insulin or albumin expression [14-16] but the hepatic and pancreatic differentiation of SGC is incomplete [16]. The treatment of mouse submandibular salivary gland cells with 5mM valproic acid for 5 days causes an increase in hepatic (*G6p*, *Alb*, *Tdo*) and pancreatic (*Ngn3*, *Pax4*, *Ins1*) markers expression levels [19]. The effect of histone deacetylase inhibitors on the differentiation potential of cells is reversible and not specific; thus, small molecules treatment is usually combined with specific differentiation cytokines.

In this study, we investigate the effect of valproic acid treatment on the hepatic differentiation of mouse submandibular salivary gland cells. For SGC hepatic differentiation, we chose a standard protocol [20, 21] including the main stages occurring during the differentiation of liver cells. Before performing the cell differentiation procedure, we treated SGC with 5mM valproic acid for 5 days and compared the effectiveness of hepatic differentiation of pretreated and intact cells. The first-passage SGC and LPC were used as controls. This approach will help to estimate the influence of valproic acid on the efficiency of salivary gland cells hepatic differentiation and will help assess the phenotypic plasticity of salivary gland cells.

## MATERIALS AND METHODS

**Animals**

C57BL/6 male mice (aged 8-15 weeks) were used. All animal experiments were performed in accordance with the Ethics Committee for Animal Research of the Koltsov Institute of Developmental Biology, Russian Academy of Sciences, as approved by the Guidelines for Humane Endpoints for Animals Used in Biomedical Research, Regulations for Laboratory Practice in the Russian Federation.

**Cell culture**

The mice were anaesthetized by injecting 300 mg/kg of chloral hydrate (Sigma) intraperitoneally. The submandibular salivary glands and the liver were excised under aseptic conditions. The organs were dissected in phosphate-buffered saline (PBS), washed twice with PBS containing 40 μg/ml gentamicin and incubated in DMEM/F12 (1:1) medium (Gibco) with 0.1% type IV collagenase (Sigma) for 30-40 min at 37°C. The cells were pipetted and passed through a filter with 40 μm pores (Corning). The cell suspensions were washed twice in DMEM/F12 culture medium using “gentle” centrifugation (2 min, 100g). The cells were plated into culture dishes (Corning) coated with collagen type I at a density of 5*103 cells per cm^2^. The cells were cultured in DMEM/F-12 (1:1) supplemented with 10% fetal bovine serum (FBS) (HyClone), 2 mM glutamine (Gibco), 1% insulin-transferrin-selenium supplement (ITS) (Invitrogen), and 10 ng/ml epidermal growth factor (EGF) (Gibco). This standard culture medium was changed every 3 days. After the monolayer formation (on day 5-7), the cells were harvested using 0.25% trypsin/ EDTA (Gibco) and plated onto collagen type I-coated culture dishes at a density of 1*10^4^.

**Culture conditions for hepatic differentiation of salivary gland cells**

A first-passage monolayer culture of SGC was used for the hepatic differentiation (approximately 15-20 day after cell isolation). SGC were divided into two groups: one group of SGC was treated for 5 days with 1mM valproic acid in a standard culture medium (the media with VPA was changed every day). Another group of SGC was incubated in a standard culture medium for 5 days without VPA treatment (the media was changed every day). Then, VPA was removed and the standard media was substituted with hepatic differentiation media: DMEM/F-12 (1:1) supplemented with 10% FBS (HyClone), 2 mM glutamine (Gibco), 1% ITS (Invitrogen), 0.03 mM Nicotinamide (Sigma), 20 ng/ml EGF (Gibco), and 20 ng/ml hepatocyte growth factor (HGF) (Invitrogen). This day was considered as the first day of differentiation. The same differentiation procedure was performed in both groups of SGC: cells were cultured in hepatic differentiation media with 20 ng/ ml BMP2 (Invitrogen) for 5 days, then with 10 ng/ml oncostatin M (Invitrogen) and 0.1 μM dexamethasone (Sigma) for 5 days, then with 1% N2 and 1% B27 (Invitrogen) for 5 days. Media changes were performed every 3 days during the differentiation procedure.

On the 15th day of cell differentiation, the cells were analyzed under 2D and 3D culture conditions. The undifferentiated first-passage SGC and LPC were used as controls.

**Immunocytochemistry**

For immunocytochemical staining, the cells were grown as a monolayer on collagen I-coated dishes for 3 days. The cells were fixed with 4% paraformaldehyde (Sigma) for 10 min and permeabilized in PBS containing 0.1% Triton-X (Sigma) for 30 min at room temperature. Non-specific binding was blocked for 30 min with PBS containing 1% bovine serum albumin (Sigma) at room temperature. The cells were incubated with primary antibodies diluted in PBS for 1 h at 370C. The antibodies and dilutions used were as follows: anti-alpha- fetoprotein (Afp), 1:200 (R&D; MAB1368); antialbumin (Alb), 1:200 (R&D; MAB1455); anti-cytokeratin 19 (Krt19), 1:100 (AbCam; ab15463-1). Then, the cells were washed three times in PBS and incubated with secondary antibodies diluted in PBS (1:1000) for 40 min at 370C. The secondary antibodies used: Alexa Fluor® 488 donkey anti-rabbit IgG (H+L) (Invitrogen; A-21206), and Alexa Fluor® 488 goat anti-mouse IgG (H+L) (Invitrogen; A-11029). The cells were washed three times for 10 min in PBS (during the last wash DAPI (Sigma) was added). The cells were viewed using the fluorescence microscope Olympus IX51. For negative controls, the secondary antibodies were used.

**Extraction of total ribonucleic acid (RNA)**

The extraction of total RNA was performed using the AllPrep DNA/RNA Mini Kit (Qiagen) according to the manufacturer’s protocol. RNA was quantified using minifluorimeter Qubit and the RNA Assay Kit (Invitrogen). The RNA was converted into complementary deoxyribonucleic acid (cDNA) using the Superscript II kit (Invitrogen) and random primers according to the manufacturer’s instructions. Five hundred nanograms of total RNA were used in the reaction.

**Quantitative RT-PCR**

**Table 1:** Primers used in qRT-PCR

Primer	Gene	Nucleotide sequence	Amplicon, bp	Melting temperature, °C
Control				
Gapdh	Glyceraldehyde-3-phosphate dehydrogenase	AGGTCGGTGTGAACGGATTTG GGGGTCGTTGATGGCAACA	95	62.6 62.6
Liver-enriched mercers				
Aat	Alpha-1-antitrypsin	CTCGTCCGCTCACTAAACAAG GCTGTCTGAGAGTCAAGGTCTT	248	60.7 61.3
Afp	Alpha-fetoprotein	CCATCACCTTTACCCAGTTTGT CCCATCGCCAGAGTTTTTCTT	101	60.2 60.6
Alb	Albumin	TGCTTTTTCCAGGGGTGTGTT TTACTTCCTGCACTAATTTGGCA	167	62.4 60.2
Krt19	Cytokeratin 19	GGGGGTTCAGTACGCATTGG GAGGACGAGGTCACGAAGC	113	62,9 62,1
Cyp7a1	Cytochrome P450, family 7, subfamily a, polypeptide 1	AACGGGTTGATTCCATACCTGG GTGGACATATTTCCCCATCAGTT	126	62.0 60.0
Cyp3a13	Cytochrome P450, family 3, subfamily a, polypeptide 13	GATTCTTGCTTACCAGAAGGGC GCCGGTTTGTGAAGGTAGAGTA	170	61,0 61,7
G6p	Glucose-6-phosphatase	CGACTCGCTATCTCCAAGTGA GGGCGTTGTCCAAACAGAAT	208	61.0 60.9
Pepck	Phosphoenolpyruvate carboxykinase1	TGACAGACTCGCCCTATGTG CCCAGTTGTTGACCAAAGGC	153	61.0 61.4
Tat	Tyrosine aminotransferase	AGCCGAATCCGAACAAAACC GCCGATAGATGGGGCATAGC	146	60.9 61.3
Tdo	Tryptophan 2,3-dioxygenase	AATCCATGACGAGCACCTATTCA TCACCTTGAGCATGTTCCTCT	140	61.4 60.8
Liver-enriched transcription factors				
Hhex1	Hematopoietically expressed homeobox 1	CGAGACTCAGAAATACCTCTCCC CTGTCCAACGCATCCTTTTTG	162	61.2 60.0
Hnf-3α	Hepatocyte nuclear factor 3α (Foxa1)	GGAGTTGAAGTCTCCAGCGTC GGGGTGATTAAAGGAGTAGTGGG	157	62.4 61.7
Hnf-3β	Hepatocyte nuclear factor 3β (Foxa2)	TCCGACTGGAGCAGCTACTAC GCGCCCACATAGGATGACA	176	62.8 61.8
Hnf-4α	Hepatocyte nuclear factor 4α	ATGCGACTCTCTAAAACCCTTG ACCTTCAGATGGGGACGTGT	135	60,0 62,7
Hnf-6	Hepatocyte nuclear factor 6	GCCCTGGAGCAAACTCAAGT TTGGACGGACGCTTATTTTCC	231	62,4 60,6
Tbx3	T-box transcription factor 3	TGGAACCCGAAGAAGACGTAG TACCCCGCTTGTGAAACTGG	84	61.2 62.1
Acinar marker				
Ptf1a	Pancreas specific transcription factor 1a	GCTACACGAATACTGCTACCG CGCAGCAATAGCTGACGTTG	134	60.3 62.0

Quantitative real-time PCR (qRT-PCR) was performed using the EVA Green kit (Syntol) and CFX96 system (BioRad). qRT-PCR reactions were performed for 40 cycles with preliminary incubation at 950C for DNA-polymerase activation. Each cycle consisted of the following steps: denaturation at 950C (30s), annealing at 57-590C (30 s), and elongation at 720C (45 s). The annealing temperature varied depending on the primer’s melting temperature (primers are listed in *Table 1* of supplemental materials). Fluorescence detection in a Fam channel and primary processing of the results were performed automatically using the system software. Samples were run in triplicate and normalized to *Gapdh*.

**DNA methylation study by bisulphite sequencing**

**Table 2:** Primers used in bisulphite sequencing

Primer	Nucleotide sequence	Amplicon, bp	Melting temperature, °C
AlbF1	TTGGTAAAGATGGTATGATTTTG	397	58,4
AlbF2	ATTTTGTAATGGGGTAGGAAT	381	58,7
AlbR	ACCACCTAAAAATTCTCAAA		57,3
Hnf-3βF	AATGTGTATTAAAAGGGAGGAAA		60,0
Hnf-3βR1	CCRAACAACCCATTTAAATAATC	378	59,2
Hnf-3βR2	CCCAAAAACCTAAAATCAAA	180	57,9
Gata4F1	TATTGAGAGTAGGGAGGAAAGA	261	60,0
Gata4F2	AGGAAAGAGAAGGAGAATAAATA	247	58,8
Gata4R	CTAACTAACCTAAAAAAATCAC		57,2
Gata6F1	ATTTAGTAGTTTGTAGAGAGTAG	405	57,1
Gata6F2	TTTYGATTTATAGTTTGGTATTTT	381	57,5
Gata6R	AATCCCTACAATCTTCTAAA		55,7
Hnf-1αF1	ATAGGGGTTTTTTTTTTTTTGGG	373	62,3
Hnf-1αF2	GGGTGTAGTGATTTATTTTA	325	55,3
Hnf-1αR	ACTTTAAACTTCAACCTTAC		56,7
Hnf-4αF	TTTGGTTTTTATAGGTATTAGGT		58,9
Hnf-4αR1	CTCTTTCTTTCTTTCTTTCTTTC	399	59,2
Hnf-4αR2	CTTTCTTTCTTTCTTTCTTTCTTTC	365	60,8
Hnf-6F	TTTTTTYGGTTTATTTGTGTTGG		60,1
Hnf-6R1	ATATCTTACCTTCTCTCTTACT	390	56,8
Hnf-6R2	TTCCCCTCTATCTTTTTTTTTTC	363	60,6

DNA was isolated and purified from frozen cells with the GenElute Mammalian Genomic DNA Miniprep Kit (Sigma-Aldrich G1N70) by the method recommended by the supplier. After DNA quantity measurement with a Qubit fluorometer (Invitrogen), it was sodium bisulphite modified with the EpiTect Bisulfite Kit (Qiagen) by the method recommended by the supplier. The primers for PCR amplification of modified DNA were constructed with the aid of the online service BiSearch (http://bisearch.enzim.hu/?m=search). The list of the primers used is in *Table 2* of supplemental materials. The target regions were chosen to contain a transcription initiation site proximal to CpG islands or immediately preceding the transcription initiation site for genes devoid of CpG islands. Two-step PCR amplification was carried out using 2 μl of the primary PCR mix as a matrix for the second PCR step and changing one of the primers used in primer PCR for a more internal one. The real-time detection system DT-322 (DNA-Technologiya, Moscow, Russia) and qPCRmix- HS SYBR+ROX kit (Evrogen, Moscow, Russia) were used. Amplification conditions were 95o-5 min for DNA initial denaturation and Taq DNA polymerase, 40 cycles of [95°-30 sec – 52-56° (T_m_ minus 3°)-30 sec – 72°-45 sec], final elongation – 72°-2 min. The final PCR mixes were fractionated by 2% agarose gel electrophoresis, discrete bands of the expected length were quickly cut out under “long” (312 nm) UV light, and DNA was extracted with the GenElute Gel Extraction Kit (Sigma-Aldrich NA1111-1KT). Sequencing of the PCR fragments obtained was done with an ABI PRISM® BigDye™ Terminator v. 3.1 kit on an Applied Biosystems 3730 DNA Analyzer. The results of the visualization and their export to a fasts format were done with the Sequence Scanner Software v1.0 (http://www.appliedbiosystems.com/absite/us/en/home/support/software-community/free-ab-software.html). DNA methylation patterns were obtained with the aid of the online service Meth Tools 2.0 (http://194.167.139.26/ methtools/MethTools2_submit.html) [22].

**Histone H3 methylation analysis**

ChIP grade Abcam antibodies (H3K4me3 – ab1012, H3K9me3 – ab8898, H3K27me3 – ab6002) and the Imprint Chromatin Immunoprecipitation Kit (Sigma- Aldrich CHP1) were used for chromatin immunoprecipitation and subsequent fractionation by the method recommended by the supplier of the kit. The resulting methylated H3-associated DNA fractions were whole-genome amplified with the REPLI-g UltarFast Mini kit (Qiagen 150033) by the method recommended by the supplier. The relative content of the target gene sequences was estimated by quantitative PCR using the real-time detection system DT-322 (DNA-Technologiya, Moscow, Russia) and the qPCRmix- HS SYBR+ROX kit (Evrogen, Moscow, Russia). 50 ng of each methylated H3-associated DNA fraction was used as a matrix for PCR amplification. The primers were chosen to amplify the target gene sequences immediately preceding the transcription initiation site (the list of primers is in *table 3* of supplemental materials). All the data obtained were normalized to those of the DNA samples obtained from the aliquots of input unfractionated chromatin.

**Table 3:** Primers used in histone H3 methylation analysis

Primer	Nucleotide sequence
AlbF	GGGGTAGGAACCAATGAAATG
AlbR	GAGGAGGAGGAGAAAGGTTA
Hnf-3βF	CACCTGCTTGTTGTTTTGAC
Hnf-3βR	AGTCCCTTCCTTTACGTCCA
Gata4F	TTGGGGGAGCTTTGGGAAGA
Gata4R	GGAAAAGAGCAGGGACTCGG
Gata6F	TACCACCACCACCATCACCAT
Gata6R	TCTGATCTTTACCTGTGCTG
Hnf-1αF	TGATGTTGGGCTAGGACTGA
Hnf-1αR	CAATTGGGAGTGAGCAGAAG
Hnf-4αF	AGACAGGGTGGATAGATAGC
Hnf-4αR	GACAGTGTGAGTATGTGTGCAG
Hnf-6F	CCACCACCTACACTACCTTA
Hnf-6R	GGTTATTCATAGAGGCCAGC

**Urea production analysis**

Analysis of the cell’s ability to produce urea was performed under 3D culture conditions in a collagen gel. The collagen gel was prepared using collagen of rat tails dissolved in 0.1% sterile acetic acid to a concentration of 5 mg/ml.

Before the gel preparation, all materials were cooled down to +4°C. The components were mixed in the following order: 0.34 M NaOH (Sigma) to a concentration of 0.023 mM, 7.5% Na_2_CO_3_ (PanEco) to a concentration of 0.26%, 10× DMEM (Sigma) to a concentration of 1×, glutamine (Gibco) to a concentration of 2 mM, HEPES (Gibco) to a concentration of 1%, fetal bovine serum (HyClone) to a concentration of 10%, and then the collagen in acetic acid to a concentration of collagen of 2%. The cell suspensions in a small volume of PBS were added in the last to a concentration of cells of 1×10^6^ per 1 ml of the gel. The mixture was stirred and placed into 35 mm Petri dishes (2 ml per dish). The Petri dishes were kept in a CO_2_ incubator at 37°C for 30 min until complete polymerization of the gel. After the gel polymerization, 2 ml of the culture medium was added into each dish. The gel was placed then into a CO_2_ incubator (zero hour of the gel preparation) and kept under standard culture conditions.

The amount of produced cell urea was determined in the culture medium using the Urea Assay Kit (Bio- Vision) in accordance with the manufacturer’s recommendations. The samples of the medium were collected on days 1, 5, and 10 of cell incubation in the gel. The medium was changed to fresh 24 hours prior to sampling. The amount of urea was determined by the intensity of the chromogenic reaction on the Start Fax 2100 microplate reader (Awareness Technology Inc).

**Statistical analysis**

All experiments were performed using cell cultures obtained from three animals with at least three repeats in each culture. Data were analyzed using Student’s t-test. A p value of 0.05 was considered significant.

## RESULTS

**Immunocytochemical and qRT-PCR analyses confirmed the increased expression of hepatocyte markers and decreased expression of ductal markers in SGC after VPA exposure**

On the 15th day of hepatic differentiation, the cells were analyzed by immunostaining with antibodies to alpha-fetoprotein (a marker of undifferentiated liver), albumin (a marker of hepatocytes), and cytokeratin 19 (a marker of ductal cells), as well as by qRT-PCR with the primers listed in *Table 1*. The differentiated SGC pretreated with VPA were named SGC-VPA-diff, and differentiated SGC without VPA exposure were named SGC-diff. For the control of cell differentiation, we used undifferentiated first-passage SGC and LPC.

**Fig. 1:**
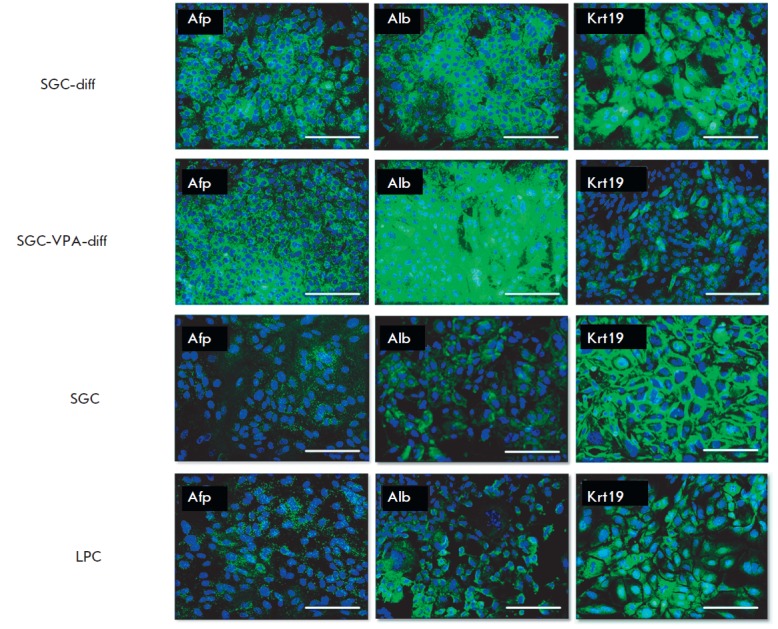
Immunocytochemical analysis of hepatic differentiation, fluorescent microscopy. Cell nuclei were stained with DAPI (blue), the antigens were detected with Alexa Fluor 488-conjugated antibodies (green), scale bars = 100 μm

In the first-passage SGC and LPC cultures, expression of alpha-fetoprotein, albumin (weakly), and cytokeratin 19 were observed (*Fig. 1*). In LPC, cytokeratin 19 was localized near the nucleus and in SGC it was also detected near the cell membrane.

In differentiated SGC, an increase in alpha-fetoprotein and albumin expression was observed; what is more, in SGC-VPA-diff, the albumin expression level was higher (*Fig. 1*). The change of cellular localization of the ductal marker cytokeratin 19 occurred during hepatic differentiation: in SGC-diff, as well as in SGCVPA- diff, the loss of cytokeratin 19 localization near the cell membrane was observed. Furthermore, in the SGC-VPA-diff culture a decrease in the cytokeratin 19 expression level was detected.

**Fig. 2:**
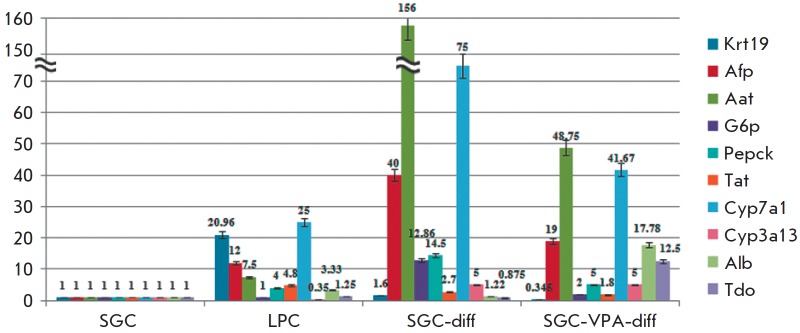
qRTPCR analysis of hepatic cell markers gene expression

**Fig. 3:**
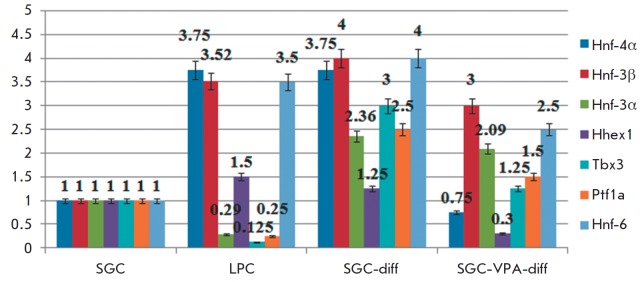
qRT-PCR analysis of liver- enriched transcription factors gene expression

The qRT-PCR analysis confirmed the results obtained using immunocytochemistry. For a more convenient interpretation of the results, we considered the gene expression levels in first-passage SGC to be “one” (after the normalization to *Gapdh *in each culture) and the expression levels of corresponding genes in other cultures were measured relative to those determined in the first-passage SGC (*Fig. 2*, *Fig. 3*).

In SGC-diff, a significant increase in the expression of early differentiation markers (*Aat*, *Afp*) was observed (*Fig. 2*). The expression of the hepatocyte markers *G6p*, *Pepck*, *Tat, *and *Cyp3a13 *also increased, but there was not significant change in *Alb *and *Tdo *expression. In SGC-VPA-diff, the expression increase in early differentiation markers (*Aat*, *Afp*) is lower than in SGC-diff; however, the expression level of *Alb *and *Tdo* increased significantly compared to SGC-diff (*Fig. 2*). The expression of ductal marker *Krt19 *is 4.6-fold lower in SGC-VPA-diff than in the SGC culture. The expression level of ductal cytochrome *P450 7a1 *also increased during hepatic differentiation; but in SGC-VPA-diff, to a lesser degree (*Fig. 2*).

The expression of liver-enriched transcription factors was also analyzed. In the first-passage LPC culture, the mRNA expression levels of the hepatocyte nuclear factors *Hnf-3β*, *Hnf-4α, *and *Hnf-6 *are about 3.5-fold higher than in SGC, but *Hnf-3α *expression is about 3.4-fold higher in the SGC culture (*Fig. 3*). After hepatic differentiation of SGC, the gene expression levels of hepatocyte nuclear factors (*Hnf-3β*, *Hnf-4α *and* Hnf-6*) increased to values observed in the LPC culture. However, the expression levels of the early hepatic differentiation gene (*Tbx3*), as well as the transcription factors involved in ductal (*Hhex1*) and acinar (*Ptf1a*) formation, increased too in SGC-diff (*Fig. 3*). Valproic acid had an ambiguous effect on the transcription factors’ gene expression: on the one hand, there was a decrease in the *Hhex1 *expression level; furthermore,* Tbx3 *and *Ptf1a *expression increased slightly. On the other hand, *Hnf-4α *expression did not increase in SGCVPA- diff (*Fig. 3*).

In general, expression of the transcription factors required for hepatic differentiation and early hepatocyte markers (*Afp *and *Aat*) is significantly increased after differentiation of mouse submandibular salivary gland cells. Increase in later differentiation markers expression (*G6p*, *Pepck*, *Tat*, *Alb, *and *Tdo*) is less pronounced. Thus, under 2D cultivation conditions, initiation and initial stages of hepatic differentiation occur. The effect of VPA treatment on the SGC differentiation is ambiguous: on the one hand, reduction of the ductal markers expression (*Krt19 *and cytochrome *P450 7a1*) and increase in the hepatocyte markers expression – *Alb* and *Tdo *is observed, which may indicate an increase in differentiation specificity. On the other hand, the expression levels of the early hepatic markers in SGCVPA- diff are usually lower than in SGC-diff. It is possible that SGC-VPA-diff is at a later stage of hepatic differentiation, which is characterized by a low level of early differentiation markers expression. Furthermore, it is known that the *Afp *and *Aat *genes normally express in salivary glands cells, and that the expression of *Aat *in ducts of the salivary glands increases during their development and differentiation [23, 24]. Initially, the SGC culture consists of undifferentiated cells. During hepatic differentiation later differentiation stages typical of the salivary gland may emerge. Thus, the observed difference in *Afp *and *Aat *gene expression for SGC-diff and SGC-VPA-diff may be a result of differentiation, characteristic for salivary glands. In this case, the decrease in the expression of these genes in SGC-VPA-diff may indicate increased specificity of hepatic differentiation and decreased lineage commitment inherent to salivary glands.

**DNA methylation analysis revealed no significant changes in the methylation of the promoter regions of liver-enriched genes in salivary gland cells**

**Fig. 4:**
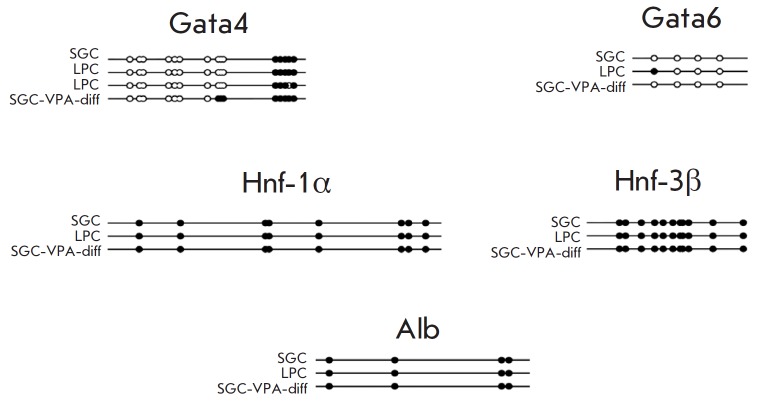
Analysis of promoter region DNA methylation pattern of liver-enriched genes. Empty circles correspond to nonmethylated CpG dinucleotides, solid circles – to methylated

The DNA methylation pattern of liver-enriched genes was analyzed for SGC, LPC, and SGC-VPA-diff cultures at the first passage by bisulfite sequencing. CpG-islands near the transcription initiation point were analyzed; and in the case of island absence – in the area immediately preceding the transcription initiation point. In most cases, the methylation of these areas is most important for gene expression. In a graphic form, generated by the online service Meth Tools 2.0, the DNA methylation pattern is shown in *Fig. 4*.

It was shown that the DNA methylation pattern is similar in all three cultures. Part of the *Gata4 *gene CpG-island, located in the promoter region, is virtually not methylated. Methylated cytosines were located below the transcription initiation point. It is possible that the *Gata4 *gene is in active or preactivated state in all three cultures. *Gata6 *is hardly methylated, and it is possible that this gene is active or is in the preactivated state in all cultures. The promoter region of the *Hnf-1α* gene is strongly methylated in all cultures, but this area is not a CpG island but sporadic CpG-site. So it is unclear how methylation affects *Hnf-1α *expression. The* Hnf-3β *gene is strongly methylated in the same manner in all three cultures; therefore, we can talk about its stable repression. But the CpG-island located at the beginning of the coding sequence is not in the promoter region. There are cases when such methylation does not prevent transcription. The *Alb *gene is methylated quite and almost to the same degree in all cultures, which may indicate its stable repression in all three cell cultures. Another possibility is that methylation of this area may be unessential for gene transcription.

Thus, for the most investigated genes no significant differences in DNA methylation patterns were observed in all cell cultures. Apparently, specific transcription control of these genes is carried out in the cells at the expense of other epigenetic modifications (possibly, histone modifications).

**Valproic acid treatment and subsequent differentiation of salivary gland cells changes the histone H3 methylation in the chromatin areas associated with liver-enriched genes**

Histone methylation plays one of the most important roles in the epigenetic regulation of transcription. Analyses of histone H3 methylation were performed on the following positions: H3K4me3 - signal most clearly correlating with the promoter transcriptional activity; H3K9me3 - signal correlating with inactivation of genes by a heterochromatization mechanism; and H3K27me3 - signal by which the polycomb repressive complex 2 (PRC2) produces an inactivating effect on the gene activity.

**Fig. 5:**
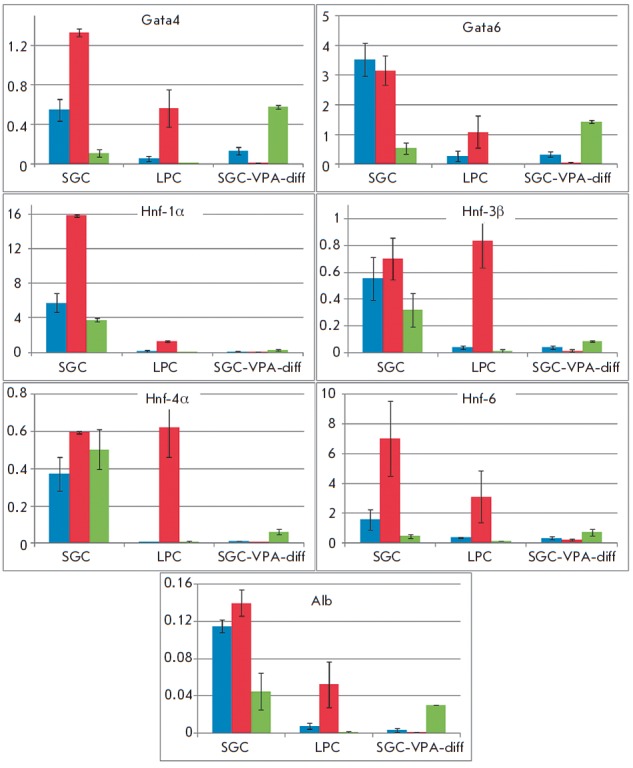
Analysis of the histone H3 methylation in the cell cultures at the first passage. Blue color shows H3 histone methylation in the H3K4me3 position, red color – H3K9me3, green color – H3K27me3 position

It was shown that histone methylation of the early endoderm genes Gata4 and Gata6 is generally similar in SGC cultures (*Fig. 5*). Compared to the control LPC, in first-passage salivary gland cells, H3 histone of these genes is methylated higher on the H3K9me3 position, which indicates their heterochromatin inactivation. At the same time, in SGC, histone methylation is present on the H3K4me3 position for both genes. The low expression levels of *Gata4 *and *Gata6 *in SGC detected by the analysis of gene expression across the transcriptome [18] suggest that inactivating methylation in the H3K9me3 position in these cells is dominant. The histone methylation level of the *Gata4 *and *Gata6* genes in the H3K9me3 position is much lower in LPC, and methylation in the H3K27me3 position is virtually absent. These results correlate with the relatively higher expression of these genes in liver progenitor cells, as shown by gene expression analysis across the transcriptome. The H3K9me3 histone methylation for* Gata4 *and *Gata6 *genes is greatly reduced in SGC-diff and SGC-VPA-diff. At the same time, H3K27me3 histone methylation of these genes slightly increases in SGC-VPA-diff. This can indicate the transcriptional activation of these genes after differentiation. However, it is also possible that in salivary gland cells secondary inactivation of these genes appears after differentiation.

The *Hnf-1α *transcription factor is strongly inactivated in SGC at the first passage by the heterochromatization mechanism. In addition, there is increased H3K27me3 methylation compared to LPC. H3K9me3 and H3K27me3 methylation are almost completely absent for this gene after differentiation. The H3K4me3 methylation level in SGC-VPA-diff is equivalent to the level in the LPC culture at the first passage. This may indicate transcription activation of this gene after hepatic differentiation.

The hepatocyte nuclear factors *Hnf-3β *and *Hnf-4α* demonstrate similar histone methylation features. In the first-passage SGC, the H3 histone methylated in the H3K9me3 and H3K27me3 positions; however, the level of H3K9me3 methylation for the *Hnf-3β *gene is slightly lower compared to LPC. These data correlate with the results obtained by RT-PCR and gene expression analysis across the transcriptome, according to which in the first-passage SGC *Hnf-3β *expression is present. The H3K9me3 methylation level for the* Hnf-4α *gene in SGC is equivalent to the level in LPC; however, the H3K27me3 methylation level is much higher in SGC. H3K9me3 methylation for the *Hnf-3β* and *Hnf-4α *genes is almost absent after differentiation, whereas H3K27me3 methylation persists at a low level. This could mean activation of these genes, which correlates with the RT-PCR results, according to which the *Hnf-3β *expression level in SGC-VPA-diff increases 3-fold in comparison to SGC.

For the *Hnf-6 *gene in SGC a high level of H3K9me3 methylation is typical. Methylation at this position is largely absent after differentiation, but it slightly increases the H3K27me3 methylation level, which could mean a secondary inactivation of this gene.

In first-passage SGC, the H3 histone of the *Alb *gene is methylated in the H3K9me3 and H3K27me3 positions. H3K9me3 methylation is almost absent, and the H3K27me3 methylation level decreases after differentiation. The H3K4me3 methylation level in SGC-VPAdiff is equivalent to that in LPC. These results could indicate transcriptional activation of this gene. According to RT-PCR results, the *Alb *mRNA expression level is low in first-passage SGC and increases 18-fold after differentiation.

Thus, these results indicate that the VPA treatment and following differentiation procedure affect the mechanisms of genome epigenetic regulation. DNA methylation of gene promoter regions differs very little in the studied cell cultures. However, the H3 histone methylation shows significant differences. The early endodermal markers *Gata4 *and *Gata6 *and hepatocyte nuclear factors *Hnf-1α *and *Hnf-6 *are inactivated by a heterochromatization mechanism in first-passage SGC. The *Hnf-3β*, *Hnf-4α, *and *Alb *genes in addition could be inactivated by the polycomb repressive complex 2 (PRC2). After differentiation in SGC, in almost all cases the removal of H3K9me3 methylation occurs. However, for the *Gata4*, *Gata6*, *Hnf-6, *and *Alb *genes secondary inactivation by H3K27me3 methylation is possible. In general, the histone methylation results correlate well with the gene expression data obtained by RT-PCR.

**Valproic acid increases urea production in mouse salivary gland cells under 3D cultivation conditions**

One of the detoxification functions of the liver is urea synthesis from ammonia, carried out by hepatocytes. Determination of the cell’s ability to produce urea is widely used as a test for hepatic differentiation efficiency estimation [25]. To assess the hepatic differentiation efficiency of SGC *in vitro, *we analyzed urea production by cells under 3D cultivation conditions (in the collagen gel). The undifferentiated first-passage SGC and LPC were used as a control.

The first-passage SGC and LPC synthesize almost no urea under 2D cultivation conditions but acquire the ability to produce urea under 3D cultivation conditions. Increased urea production level by the studied cells in the collagen gel during the whole observation period indicates that 3D cultivation conditions promote cell differentiation.

**Fig. 6:**
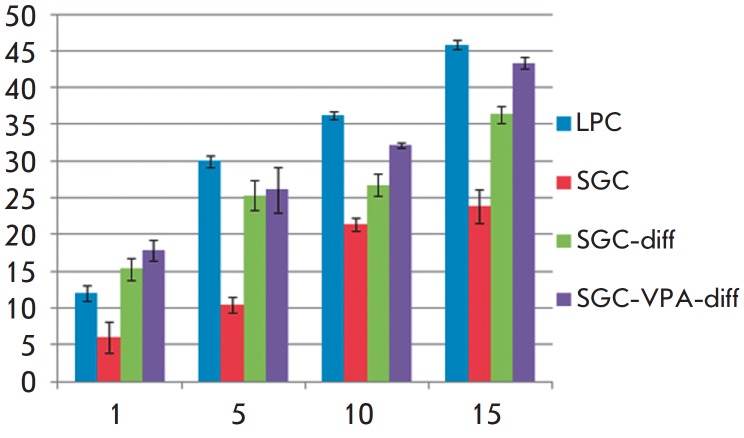
The urea production analysis by differentiated cells in comparison with first-passage SGC and LPC under 3D cultivation conditions. The X-axis value is the days of cell cultivation in the gel, the Y-axis value is the amount of urea in the medium (mM) per 1x10^6^ cells per 24 hours

By the 15th day of cell incubation in collagen gel, the level of urea production reaches 24 mM per 1x10^6^ cells per 24 hours (*Fig. 6*). For comparison, freshly isolated mouse hepatocytes produce about 350 mM of urea per 1x106 cells per 24 hours. The high level of urea production by SGC is evidence of their considerable potential for hepatic differentiation under certain culture conditions. The LPCs actively synthesize urea under 3D conditions: by the 15th day, the urea production level by PKP is 7.6 fold lower than that of the primary culture of hepatocytes. This indicates the high hepatic differentiation ability of LPC under 3D cultivation conditions.

After hepatic differentiation, the ability of SGC to produce urea increases, and, in the case of VPA treatment, SGCs produce urea at a comparable rate with LPC. Thus, hepatic differentiation affects not only the gene expression of submandibular salivary gland cells, but also the functional characteristics of these cells. Hepatic cell differentiation of SGC yields cells capable of performing some of the functions of hepatocytes. VPA can increase cell differentiation efficiency.

## DISCUSSION

Using small molecules that can affect the epigenetic regulation of gene expression and cause an increase in differentiation efficiency is a promising approach in cell biology. It has been shown that valproic acid increases the differentiation efficiency of various cell types. Dong *et al*. [9] showed that VPA treatment significantly increases the efficiency of hepatic differentiation of mouse ES cells and decreases the extent of their spontaneous differentiation into bile duct structures. The authors suggest that the possible mechanism accelerating the ES cells differentiation could be the transition of the cell cycle in the G0/G1 phase, which occurs after VPA treatment. Deceleration of the cell cycle contributes to the loss of pluripotency and differentiation of ES cells.

VPA can also increase the differentiation efficiency of committed cells. VPA exposure for 72 hours at 5 mM in human bone marrow cells increases H3 and H4 histone acetylation, which promotes DNA demethylation of these cells [10]. During the subsequent hepatic differentiation, human bone marrow cells express albumin and store glycogen more efficiently than cells not exposed to VPA. Differentiated bone marrow MSCs were able to produce urea; what is more, after VPA treatment urea synthesis was about 1.5 fold higher [10]. The authors suggested that the increased differentiation efficiency of bone marrow cells is due to demethylation of the genes involved in hepatic differentiation.

In addition, the histone deacetylase inhibitor - valproic acid can cause active DNA demethylation in a DNA-replication-independent manner [7]. This effect may also increase the transcriptional activity of genes. Furthermore, VPA activates the *Wnt *family genes [26]. It has been shown that the Wnt/β-catenin signaling pathway is required for the formation and differentiation of endodermal cells of the pancreas and liver [27, 28].

Our study of submandibular salivary gland cells in comparison to liver progenitor cells in mice provides insight into the ability of SGC for hepatic differentiation and the effect of valproic acid treatment on the differentiation efficiency. We have shown that the initiation and initial stages of SGC hepatic differentiation is implemented effectively, affecting a wide range of transcription factors and various liver-enriched markers. However, under 2D cultivation conditions, it is quite difficult to achieve terminal differentiation stages. For this reason, we evaluated the efficacy of differentiation under 3D cultivation conditions. Cultivation of cells in a collagen gel promotes an expansion of their morphogenetic and differentiation potential [29, 30]. Under 3D cultivation conditions, SGC-VPA-diff produce urea at a high level, comparable to the control LPC. This indicates an effective hepatic differentiation of SGC, which affects the functional features of these cells.

Hepatic differentiation has almost no effect on the DNA methylation of the genes’ promoter regions, which is not surprising given the conservative mechanism of DNA methylation. However, VPA treatment and subsequent differentiation to a large extent influence H3 histone methylation. It was shown that histone methylation in most cases is higher in control SGC and usually lower in differentiated SGC. In general, this can be explained by the progenitor nature of SGC: many genes in progenitor cells have a bivalent configuration, i.e., enriched both in activating and inhibiting histone modifications. This allows them to differentiate into different directions. The histone methylation results correlate well with the results of gene expression in the studied cells.

Most likely, there are several mechanisms of VPA influence on the cell differentiation potential. The efficiency of cell differentiation depends not only on the target cell markers acquisition, but also on the loss of the differentiation features of the initial cell line. As is known from the experience of cell reprogramming, the committed cells acquire the differentiation markers of target cells easier, while the loss of parental cell line markers occurs more slowly [31]. According to our results, VPA can reduce the expression of a number of markers characteristic of initial SGC. One of the possible mechanisms of VPA influence on the differentiation efficiency is that VPA promotes the erasing of the parental cell line epigenetic program that accelerates differentiation. In addition, the possible mechanism of VPA influence on the differentiation efficiency could be H3 histone modifications of target genes and increased accessibility of these genes for the growth factors and cytokines used in the differentiation protocol.

## CONCLUSION

The obtained results allow us to conclude that mouse submandibular salivary gland cells show significant phenotypic plasticity and are able to differentiate in the hepatic direction. Valproic acid affects the epigenetic regulation of gene expression by histone modifications and can increase the specificity and efficiency of hepatic differentiation for these cells. The possible mechanism of valproic acid influence on the differentiation efficiency could consist in erasing the parental cell line differentiation features or/and in facilitating the accessibility of target genes for the cytokines and growth factors used during hepatic differentiation.

## Abbreviations

DAPI: 4’,6-diamidino-2-phenylindole
EGF: Epidermal Growth Factor
ITS: Insulin-Transferrin-Selenium
LPC: liver progenitor cells
qRT-PCR: quantitative real-time polymerase chain reaction
SGC: salivary gland cells
SGC-diff: differentiated salivary gland cells
SGC-VPA-diff: differentiated salivary gland cells pretreated with valproic acid
VPA: valproic acid
